# Pathophysiological pathway differences in children who present with COVID-19 ARDS compared to COVID -19 induced MIS-C

**DOI:** 10.1038/s41467-022-29951-9

**Published:** 2022-05-02

**Authors:** Conor McCafferty, Tengyi Cai, Delphine Borgel, Dominique Lasne, Sylvain Renolleau, Meryl Vedrenne-Cloquet, Damien Bonnet, Jemma Wu, Thiri Zaw, Atul Bhatnagar, Xiaomin Song, Suelyn Van Den Helm, Natasha Letunica, Chantal Attard, Vasiliki Karlaftis, Slavica Praporski, Vera Ignjatovic, Paul Monagle

**Affiliations:** 1 Haematology Research, Murdoch Children’s Research Institute, Melbourne, VIC Australia; 2 Department of Paediatrics, University of Melbourne, Melbourne, VIC Australia; 3 Department of Biological Hematology, Necker Hospital, AP-HP, F-75015 Paris, France; 4 Pediatric Intensive Care Unit, Necker Hospital AP-HP, F-75015 Paris, France; 5 M3C-Necker, Congenital and Pediatric Cardiology, Necker Hospital, AP-HP, F-75015 Paris, France; 6 Australian Proteome Analysis Facility, Sydney, NSW Australia; 7 Department of Clinical Haematology, Royal Children’s Hospital, Melbourne, VIC Australia; 8 Kids Cancer Centre, Sydney Children’s Hospital, Randwick, NSW Australia

**Keywords:** Proteomics, Paediatric research

## Abstract

COVID-19 has infected more than 275 million worldwide (at the beginning of 2022). Children appear less susceptible to COVID-19 and present with milder symptoms. Cases of children with COVID-19 developing clinical features of Kawasaki-disease have been described. Here we utilise Mass Spectrometry proteomics to determine the plasma proteins expressed in healthy children pre-pandemic, children with multisystem inflammatory syndrome (MIS-C) and children with COVID-19 induced ARDS. Pathway analyses were performed to determine the affected pathways. 76 proteins are differentially expressed across the groups, with 85 and 52 proteins specific to MIS-C and COVID-19 ARDS, respectively. Complement and coagulation activation are implicated in these clinical phenotypes, however there was significant contribution of FcGR and BCR activation in MIS-C and scavenging of haem and retinoid metabolism in COVID-19 ARDS. We show global proteomic differences in MIS-C and COVID-ARDS, although both show complement and coagulation dysregulation. The results contribute to our understanding of MIS-C and COVID-19 ARDS in children.

## Abstract (web summary)

While rare, SARS-CoV-2-infected children can develop severe COVID-19 (ARDS) or inflammatory syndrome (MIS-C). Here, the authors use proteomics to characterize hundreds of blood proteins and identify key biological pathways that differentiate MIS-C and ARDS.

## Introduction

The COVID-19 pandemic has caused substantial damage to population health worldwide, infecting more than 275 million individuals and killing at least 2 million, at the beginning of 2022^1^. The clinical spectrum of COVID-19 ranges from asymptomatic or mild upper respiratory tract infection symptoms to severe pneumonia with acute respiratory distress syndrome (ARDS) culminating in respiratory failure and death^2–5^.

Compared with adults and the elderly, children appear to be less susceptible to SARS-CoV-2 infection and generally present with milder symptoms^2,6–8^. Only 1.7% of reported paediatric cases of COVID-19 included admission to the ICU^9^. COVID-19 associated acute respiratory distress syndrome (COVID-19 ARDS) is one of the major manifestations of the severe cases, characterised by hypoxemic respiratory failure with bilateral lung infiltrate, as well as multi-organ dysfunction and extensive microthrombus formation^10^. In addition, a minority of children with COVID-19 present with an unexplained multisystem inflammatory syndrome termed ‘multisystem inflammatory syndrome in children’ or MIS-C^11–20^, also known as the Pediatric Multisystem Inflammatory Syndrome Temporally associated with SARS-CoV-2. Signs of MIS-C manifested 2–4 weeks after the SARS-CoV-2 infection, showing similar clinical features to Kawasaki disease and toxic shock syndrome^14,16,20^. Symptoms associated with MIS-C include persistent fever, asthenia, systemic hypotension, gastrointestinal symptoms (e.g. abdominal pain, vomiting, or diarrhoea), skin rash, conjunctivitis, cheilitis, severe complications such as impaired cardiac function, as well as shock, myocarditis, coronary artery dilatation and aneurysm^12,15–17,19,21,22^.

Patterns of increased inflammatory/cardiac markers C-reactive protein, D-dimer, IL-6, ferritin, troponin-I and NT-proBNP have been detected in MIS-C, but a lack of confirmatory laboratory testing and overlapping clinical manifestations makes it difficult to quickly identify patients with MIS-C^12,21,22^. Whilst it appears that inflammation is the underlying cause of MIS-C, the specific pathophysiological mechanism of this clinical phenotype, as well as COVID-19 ARDS in children is unclear.

To date, proteomic analysis of blood samples in the context of COVID-19 has focused on the effect of the disease on proteome-wide expression and search for biomarkers in the adult population^23,24^.

Here, we characterise the underlying mechanisms associated with severe COVID-19 phenotypes in children (MIS-C and COVID-19 ARDS) and how their plasma proteomic pathways differ from healthy children. We hypothesised that there would be distinct proteomic differences between MIS-C and COVID-19 ARDS, reflecting their clinical presentation.

## Results

### Sample characteristics

54 samples were collected for this study, with patient demographics summarised in Table 1. Sample size was limited by availability of MIS-C and COVID-19 ARDS patients, whose clinical characteristics are listed in Supplementary Data 1. SWATH acquisition of the 54 samples were obtained, and then matched to the local assay library for quantitation of peptide areas, resulting in identification of 319 proteins. SWATH-MS passed quality control, interquartile coefficient of variance (CV) between pooled sample, commercial control and technical replicates were below 10% with a median CV of 3%.

**Table 1:** Participant demographics.

Group	Total number	Sex	Age (mean ± SD)
Healthy Children	20	12 M/8 F	8.42 ± 6.51
MIS-C Patients	29	13 M/16 F	4.56 ± 2.56
COVID-19 ARDS Patients	5	3 M/2 F	9.38 ± 3.04
*P* values (based on two-sided t-test)		0.18	0.48

No MIS-C patients had any pre-existing conditions. 1 COVID-19 ARDS patient had sickle-cell anaemia and another COVID-19 ARDS patient had leukaemia.

At the time of blood collection, all but two MIS-C patients had received IVIG in the past 48 h, and eight had received steroid treatment in the past 48 h. At the time of blood collection two COVID-19 ARDS patients did receive IVIG and two were already on steroid treatment.

### Overall differences across the Healthy, COVID-19 ARDS and MIS-C Groups

**Fig. 1:**
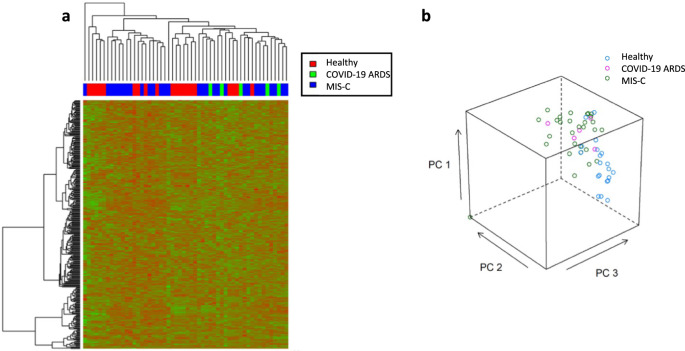
Hierarchical clustering of all detected proteins. **a** Unsupervised hierarchical clustering analysis for the 319 protein identifications using the local assay library. Relative expression patterns obtained using Euclidean distance; Green: proteins with decreased expression; Red: proteins with increased expression; **b** Principal Component Analysis of data from **a**. The axis of PC1, PC2 and PC3 represented the first three principal components.

Unsupervised hierarchical clustering of the 319 identified plasma proteins showed minimal group clustering (Fig. 1a) across the three groups, a finding supported by the principal component analysis (PCA) showing minimal separation between the groups (Fig. 1b).

**Fig. 2:**
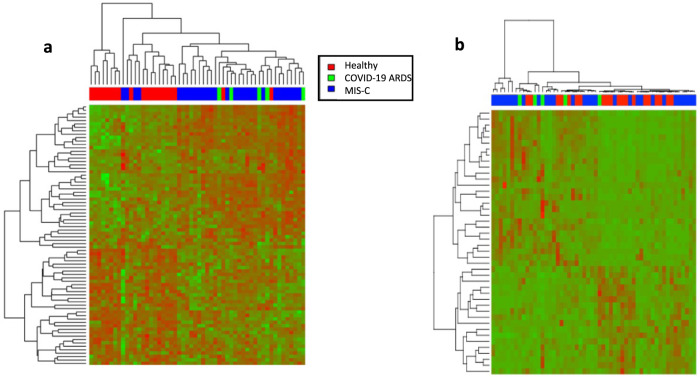
Overall differentially expressed proteins. **a** Heatmap of the 76 differentially expressed proteins from the local assay library identified using unadjusted *p* value and fold-change with two-sided *t* test. The clustering patterns were obtained using a Euclidean-based distance and complete linkage; Green: proteins with decreased expression; Red: proteins with increased expression; **b** Heatmap of the 44 differentially expressed proteins from the local assay library identified using *p* values adjusted for multiple comparisons by using two-sided *t* test with Benjamini-Hochberg correction.

Using unadjusted *p* value and fold-change criteria a total of 76 proteins were differentially expressed across the Healthy, COVID-19 ARDS and MIS-C Groups, which were ranked by maximum fold changes for Ratio of largest/lowest group average in Supplementary Data 2. Using Benjamini-Hochberg adjusted *p* values there were 44 differentially expressed proteins across Healthy, COVID-19 ARDS and MIS-C Groups. The maximum fold change ranged from 18.50 folds (Transmembrane protein 25, Uniprot Accession. Q86YD3) down to 1.53 folds (Apolipoprotein D, Uniprot Accession. P05090). The resulting hierarchical clustering of the 76 proteins showed a more defined group distribution of the three participant groups (Fig. 2). Based on the hierarchical clustering, the majority of the Healthy Group samples clustered together, albeit with some minor overlap from the MIS-C Group. Samples from the COVID-19 ARDS group and samples from the MIS-C group did not separate distinctly, but were generally separated from the healthy participants, showing the overall proteomic differences among the three groups.

### COVID-19 ARDS Group Vs Healthy Group

52 proteins were differentially expressed between the Healthy and COVID-19 ARDS groups (Supplementary Data 3) using the criteria of unadjusted *p* values and fold-change. Compared with the Healthy Group, 28 of the 52 proteins were increased in the COVID-19 ARDS Group. Among these 28 increased proteins, the minimum fold change of 1.62 was observed for Progranulin (Uniprot Accession. P28799), ranging up to a 10.20-fold increase for Immunoglobulin kappa variable 3D-20 (Uniprot Accession. A0A0C4DH25). While 24 proteins were decreased in the COVID-19 ARDS Group and the minimum and maximum fold changes proteins were Vitamin D-binding protein (Uniprot Accession. Q6LDC6) and Transmembrane protein 25 (Uniprot Accession. Q86YD3), with 1.74- and 16.47-fold changes observed, respectively. Using the criteria of Benjamini-Hochberg adjusted *p* values, 17 proteins were significantly different between the study groups.

**Fig. 3:**
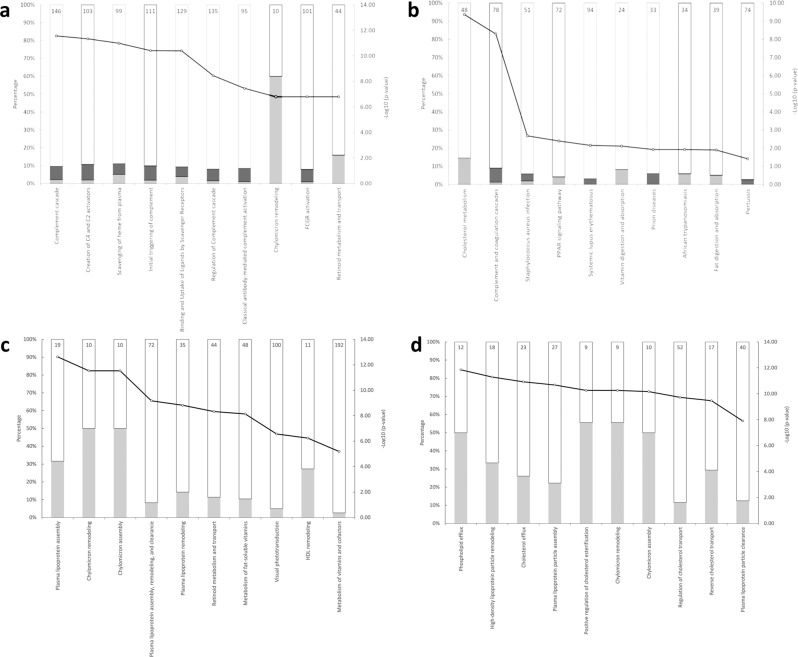
Enriched pathways between COVID-19 ARDS and Healthy Group. Top 10 enriched pathways for the 52 differentially expressed proteins based on the unadjusted *p* value and fold-change comparison between COVID-19 ARDS Group and Healthy Group, ranked in increasing order of their *p* values (determined using two-sided *t* test) from left-to-right. (**a** Reactome pathway analysis; **b** STRING pathway analysis). Top 10 enriched pathways for the 17 differentially expressed proteins based on the adjusted *p* value (using two-sided *t* test adjusted for multiple comparison with Benjamini-Hochberg correction) comparison between COVID-19 ARDS Group and Healthy Group, ranked in increasing order of their *p* values from left-to-right. (**c** Reactome pathway analysis; **d** STRING pathway analysis). The size of the bar in each graph indicates the proportion of proteins in that pathway that are up- or down-regulated in our study, the number reported at the top of each bar is the specific number of proteins in that pathway affected. Dark grey: proteins with relative increased expression in COVID-19 ARDS Group; Light grey: proteins with relative decreased expression in COVID-19 ARDS Group. Black line with a white circle: -Log10 (*p* value). Indicated pathways suggest biological pathways that are most impacted as a result of COVID-19 ARDS.

The pathway topology analysis of these 52 proteins based on the Reactome and STRING is shown in Fig. 3. The top three pathways in order of significance were, Reactome: Complement cascade, Creation of C4 and C2 activators and scavenging of haem from plasma; STRING: Cholesterol metabolism, Complement and coagulation cascades and Vitamin digestion and absorption. Pathway topology analysis of the 17 proteins selected using stricter criteria were, Reactome: Plasma lipoprotein assembly, chylomicron remodelling and chylomicron assembly; STRING: phospholipid efflux, high-density lipoprotein particle remodelling and cholesterol efflux.

### MIS-C Group Vs Healthy Group

85 proteins were differentially expressed between the MIS-C and Healthy groups (Supplementary Data 4). Compared with the healthy Group, 52 out of 85 proteins were increased in the MIS-C Group. Among the 52 upregulated proteins, the minimum fold change of 1.50 was observed for Leucine-rich alpha-2-glycoprotein (Uniprot Accession. P02750), ranging up to 13.08-fold increase for Immunoglobulin kappa variable 3D-20 (Uniprot Accession. A0A0C4DH25). For the 33 proteins decreased in the MIS-C Group, the fold changes ranged from 1.53 in Apolipoprotein D (Uniprot Accession. P05090) to 3.18 in Insulin-like growth factor II (Uniprot Accession. P01344). Using the criteria of Benjamini-Hochberg adjusted *p* values, 26 proteins were significantly different between groups.

**Fig. 4:**
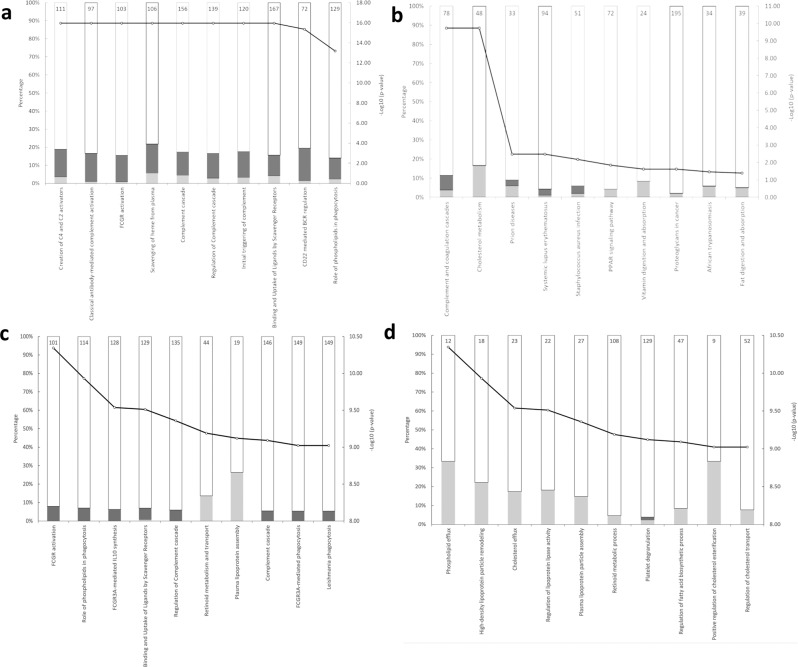
Enriched pathways between MIS-C and Healthy Group. Top ten enriched pathways for the 85 differentially expressed proteins based on the unadjusted *p* value and fold change comparison between MIS-C Group and Healthy Group, ranked in increasing order of their *p* values (determined using two-sided *t* test) from left-to-right. (**a** Reactome pathway analysis; **b** STRING pathway analysis). Top 10 enriched pathways for the 26 differentially expressed proteins based on the adjusted *p* value (using two-sided *t* test adjusted for multiple comparisons with Benjamini-Hochberg correction) comparison between MIS-C Group and Healthy Group, ranked in increasing order of their *p* values from left to right. (**c** Reactome pathway analysis; **d** STRING pathway analysis). The size of the bar in each graph indicates the proportion of proteins in that pathway that are up- or down-regulated in our study, the number reported at the top of each bar is the specific number of proteins in that pathway affected. Light grey: proteins with relative decreased expression in MIS-C Group; Dark grey: proteins with relative increased expression in MIS-C Group; Black line with a white circle: -Log10 (*p* value); Number in each column represents the total proteins number of the pathway. Indicated pathways suggest biological pathways that are most impacted as a result of COVID-19 ARDS.

The pathway topology analysis of these 85 proteins based on the Reactome and STRING were shown in Fig. 4. The top three pathways in order of significance were, Reactome: Creation of C4 and C2 activators, Classical antibody-mediated complement activation and Fc $\gamma$ Receptor (FCGR) activation; STRING: Cholesterol metabolism, Complement and coagulation cascades and prion disease. Pathway topology analysis of the 26 proteins selected using stricter criteria was, Reactome: FCGR Activation, role of phospholipids in phagocytosis and FCGR3A-mediated IL-10 synthesis; STRING: Phospholipid efflux, high-density lipoprotein particle remodelling and cholesterol efflux.

## Discussion

This plasma proteomic evidence shows that the pathogenesis of MIS-C and COVID-19 ARDS in children may be associated with changes in the complement activation and coagulation pathways. In addition to those two main common pathways, the MIS-C and COVID-ARDS differed in that the MIS-C phenotype was also associated with changes in the FcGR and BCR pathways, as well as uptake of ligands, whilst changes in the retinoid metabolism and haem scavenging systems are observed in the clinical phenotype in COVID-ARDS patients. An advantage of the pre-pandemic healthy controls in our study, is that we are certain our controls are not SARS-CoV-2 exposed.

Our finding that the complement system is affected in MIS-C is consistent with previous research, however, the previous study used targeted techniques to investigate specific proteins, such as C5b9, and did not analyse the proteome as a whole^25^. We provide holistic evidence of the affected complement system in MIS-C, and our proteomic approach gives us insight into the potential trigger of complement activation. The increase in Ficolin-2 that we show suggests, at least in children, that complement activation may be induced through the lectin pathway, which has been discussed in severe COVID-19 in adults^26,27^. Regarding coagulation, COVID-19 induces a hypercoagulable state in children and our findings for antithrombin-3 and fibrinogen in children are concordant with this^28^. In adults, this hypercoagulable state has been linked to higher mortality in COVID-19 patients, though it is unclear as yet if this is the case for children^29^.

The main pathways involved uniquely in MIS-C are Fc $\gamma$ Receptor (FcGR) activation and B-cell receptor (BCR) activation. FcGR receptors are crucial for an antibody-mediated immune response suggesting strong antibody involvement in development of MIS-C. Additionally, upregulated Fc receptors may be indicative of the IVIg treatment received by MIS-C patients, as this effect of IVIg has been established previously^30^.

Binding and uptake of ligands is a pathway with many functions but has long been associated with uptake of aberrant molecules involved in atherosclerosis, its appearance in our study may point towards endothelial involvement in the MIS-C phenotype. Based on clinical similarity MIS-C patients were treated similarly to Kawasaki patients and our findings that they have similar underlying pathophysiology supports a similar treatment decision^31^.

Scavenging of haem from plasma is a pathway that appears upregulated in children with COVID-19 ARDS. While it is difficult to make strong claims due to the small sample size, this pathway is expected and highly logical as free haem is a sign of haemolysis, which is associated with ARDS in other infections^32,33^. It should be noted that a single ARDS patient had sickle-cell disease, and while this may increase these pathways, no proteomic analysis to date has linked the pathways suggested with sickle-cell anaemia. We hypothesise that the lung injury in COVID-19 ARDS similarly leads to haemolysis and subsequent load on the haem scavenging system, though further testing to establish this is required^34^. Some hypotheses have suggested a connection between retinoid dysregulation and the development of ARDS from COVID-19 in adults, and our results suggest this link may also occur in children^35^. Interestingly, retinoid dysregulation has been associated with other viral infections in children, further supporting its importance to COVID-19 ARDS^36,37^.

In a small discovery-proteomics study such as this one, there is a careful balance between how strictly the false-discovery rate (FDR) is regulated, as some technique to account for multiple testing is paramount in proteomic studies^1^. For a small population study such as this, we prefer the less-strict unadjusted *p* value and fold change cut-off, however for completeness, we also show Benjamini–Hochberg adjusted *p* values which have a lower FDR. A significant limitation in interpreting the results in our COVID-19 ARDS population is the low sample size in comparison to the other groups, which reflects the rarity of COVID-19 ARDS in children in Australia. However, our study provides the basis for future studies with access to larger sample numbers to expand on our research. Our study also only uses a discovery proteomics technique and, while we have established a foundation for further research, targeted protein analysis will be required before this work could be implemented clinically.

We observed complement activation and coagulation dysregulation in children with MIS-C and COVID-ARDS with additional contribution of FcGR and BCR activation in MIS-C and we suggest the scavenging of haem and retinoid metabolism in COVID-19 ARDS. This knowledge adds to our understanding of the severe COVID-19 phenotypes in children and suggests pathophysiological mechanisms that could be the subject of specific therapies to try and improve the outcomes for these children. We establish a foundation for proteomic research in MIS-C and COVID-19 ARDS in children that should be built on in future clinical research.

## Methods

### Participants recruitment

This study was approved by the Royal Children’s Hospital Ethics in Human Research Committee, reference number 34184; and the Necker Hospital, France, reference number Assistance Publique - Hôpitaux de Paris- registration N°.2020 0428163907 and Trial Registration: NCT04420468. Written informed consent was obtained from the parent or guardians of healthy child participants and participants were not compensated for this study. Patient criteria of COVID-19 ARDS and MIS-C were respectively defined according to the interim guidance of World Health Organization for novel coronavirus^10^ and The Paediatric Acute Lung Injury Consensus Conference^38^.

Blood samples from healthy children were collected, processed and stored at −80 °C prior to the COVID-19 pandemic, from children attending day-surgery at the Royal Children’s Hospital, Australia, as part of the HAPPI Kids study^39^. Blood samples from SARS-CoV-2 infected children with MIS-C or COVID-19 ARDS were collected from children at Necker Hospital, France in 2020. Patients’ plasma samples were taken as part of routine care within the first 48 h of PICU stay, with excess being stored for research.

### Diagnostic criteria

For diagnosis of MIS-C at Necker Hospital, children had a persistent fever (>38.5 °C) for more than 3 days, and multiorgan involvement, and evidence for coagulopathy, and inflammation, and evidence for SARS-CoV-2 infection. A diagnosis of COVID-19 was given if a child tested positive to either polymerase chain reaction testing, serological testing or CT scan. Three clinical criteria among the following were required to be observed to define multiorgan involvement in this series: cervical lymphadenopathy, bulbar conjunctivitis, skin rash, erythema of oral and pharyngeal mucosa, gastrointestinal symptoms (abdominal pain, diarrhoea, vomiting), asthenia, meningismus, respiratory signs, heart failure, or cardiogenic shock. Evidence for coagulopathy was assessed by elevated D-dimers > 1000 ng/mL. Inflammation was defined as C-reactive protein > 80 mg/L.

All patients of the ARDS group included in the study had moderate to severe ARDS according to the Pediatric Acute Lung Injury Consensus Conference Group for the diagnosis of ARDS in COVID19 patients^38^ and required invasive mechanical ventilation.

Children who met the criteria for either MIS-C or COVID-19 ARDS were approached for enrolment in this study.

### Plasma sample preparation

Plasma preparation has been described elsewhere^39^. Briefly, all venous blood samples were collected into S-Monovette tubes (Sarstedt, Australia) or Vacuette (Greiner Bio-one, France) containing 1-part sodium citrate to 9-parts blood. Samples were centrifuged at 2500 *g* at room temperature for 10 min (twice) and stored at −80 °C until sample testing. To eliminate risk of handling COVID-19 samples, all plasma was added to TRIzol (Thermo Fisher, United States) in a ratio of 1-part plasma to 4-parts TRIzol.

Plasma samples from different groups of healthy children and SARS-CoV-2 infected children were separated into 9 batches in a randomised fashion. Each batch was processed individually from sample preparations all the way to SWATH data acquisitions. Furthermore, we have implemented a workflow with an additional inclusion of a control plasma standard sample, a technical replicate where one sample from each batch was randomly selected and acquired twice, as well as samples pooled from one batch in data acquisition and data analysis for assessing data quality in each batch.

Sample (350 µL) containing 70 µL of plasma and 280 µL of TRIzol was taken for protein extraction. 5 N acetic acid followed by chloroform were added with centrifugation at 12,000 × *g* for 15 minutes at 4 °C to separate into three phases. The top phase was discarded, and ethanol was added to precipitate DNA from the interphase, followed by centrifugation at 2000 × *g* for 5 min at 4 °C. Supernatant was collected and protein precipitated by addition of isopropanol. Protein pellet was collected by centrifugation and washed two times with 0.3 M guanidine hydrochloride in 95% ethanol solution, then with 100% ethanol. Protein pellet was collected by centrifugation at 7500 × g for 5 minutes at 4 °C. After a brief vacuum centrifugation, the protein pellet was dissolved in 100 µL of 4 M urea, 1% sodium deoxycholate (SDC), 100 mM triethylammonium bicarbonate (TEAB) solution, aided by brief probe sonication.

Each sample was taken and diluted (1:3) with 1% SDC, 100 mM TEAB solution. Samples were reduced with dithiothreitol (10 mM DTT) and alkylated with iodoacetamide (20 mM IAA). Sample proteolysis was performed by digestion with trypsin (1:25 ratio) at 37 °C overnight.

Following digestion, pH was adjusted to approximately three using a final concentration of ~1% FA, and each sample desalted using a Stage tip containing Styrene Divinyl Benzene (Empore SDB-RPS 47 mm extraction disk, SUPLCO). Briefly, stage tips were self-packed into pipette tips, peptides were bound to the stage-tip, washed with 0.2% trifluoracetic acid and finally eluted with 80% acetonitrile: 5% ammonium hydroxide. Peptides were dried by vacuum centrifuge and then reconstituted in 50 µL of 2% acetonitrile, 0.1% formic acid (loading buffer). For SWATH-MS data, 5 μL of digested sample from each batch was diluted with loading buffer (1:1) prior to injection.

Preparation and digestion of control plasma sample—a commercially available human plasma was used as a control plasma standard sample. Sample (ST01, 25 μL) was taken and diluted with 475 μL of 50 mM ammonium bicarbonate (ABC) solution. Sample was reduced with DTT (5 mM) and alkylated with iodoacetamide (10 mM IAA). 50 μL of reduced and alkylated solution was diluted in 50 mM ABC solution to a final concentration of ~1 μg/μL of plasma protein. The sample was then taken for digestion with trypsin (1:25 ratio) at 37 °C overnight. The digested sample was acidified and 10 μL was injected for SWATH MS analysis.

High pH Reverse Phase-HPLC—for ion library generation through high pH (HpH) fractionation, the non-depleted plasma samples from twenty-eight subjects infected with SARS-CoV-2 were pooled and fractionated by High pH reverse-phase high-pressure liquid chromatography (RP-HPLC). The sample was resuspended in mobile phase buffer A (5 mM ammonium hydroxide solution (pH 10). The composition of buffer B was 5 mM ammonia solution with 90% acetonitrile (pH 10). After sample loading and washing with 3% buffer B for 10 min at a flow rate of 300 μL/min, the buffer B concentration was increased from 3% to 30% over 55 min and then to 70% between 65 and 75 min and to 90% between 75 and 80 min. The eluent was collected every 2 min at the beginning of the gradient and at 1-min intervals for the rest of the gradient.

2D-IDA—following HpH-RP-HPLC separation, 17 fractions were concatenated (0–85 min), dried and resuspended in 30 µL of loading buffer. 10 µL/fraction was taken for 2D-IDA analysis.

### SWATH-MS

Information Dependent Acquisition and SWATH Acquisition—A 6600 TripleTOF mass spectrometer (Sciex, Framingham, MA) coupled to an Eksigent Ultra-nanoLC-1D system (Eksigent Technologies, Dublin, CA) was employed for both IDA and SWATH-MS analysis.

Peptides were loaded onto a reverse phase peptide C18 trap (1 cm × 300 µm, Prontosil 120 C18H, 5 µm (Dr. Maisch GmbH)) for pre-concentration and desalted for 3 min with the loading buffer (0.1% (v/v) formic acid) at a flow rate of 10 $\mu$L per minute. After desalting, the peptide trap was switched in-line with an analytical column (15 cm × 300 µm, 3C18-CL-120, 3 µm particles—120 Å pores (Eksigent)). Peptides were eluted and separated from the column using the buffer B (99.9% (v/v) acetonitrile, 0.1% (v/v) formic acid) gradient starting from 5% and increasing to 35% over 60 min at a flow rate of 5 $\mu$L per minute. After peptide elution, the column was flushed with 95% buffer B for 6 min and re-equilibrated with 95% buffer A (0.1% (v/v) formic acid) for 5 min before next injection. Blanks were run between samples. In IDA mode, a TOFMS survey scan was acquired at *m*/*z* 350–1500 with 0.25 s accumulation time, with the twenty most intense precursor ions (2^+^–5^+^; counts > 200) in the survey scan consecutively isolated for subsequent product ion scans. Dynamic exclusion was used with a window of 30 s. Product ion spectra were accumulated for 100 ms in the mass range *m*/*z* 100–1800 with rolling collision energy.

IDA Data Analysis—Protein identifications from 2D-IDA data were performed with ProteinPilot (v5.0, Sciex) using the Paragon algorithm in thorough mode. The search parameters were as follows: sample type: identification; cys alkylation: iodoacetamide; digestion: trypsin; instrument: TripleTOF 6600; special factors: none; species: Homo sapiens; ID focus: biological modifications. The database used was obtained from SwissProt (20,420 entries, May 2019) with the addition of a coronavirus database downloaded from UniProt (reviewed, 520 entries, October 2020). A reversed-decoy database search strategy was used with ProteinPilot, with the calculated protein FDR equalling 1%.

SWATH Library Construction—The ProteinPilot group file from the 2D-IDA search result was imported into PeakView (v2.2) (Sciex) and used as a local peptide assay library. This library contained 387 proteins identified from un-depleted plasma samples.

SWATH-MS— For SWATH-MS experiments 10 $\mu$L of each sample (5 $\mu$L of digested sample diluted with 5 $\mu$L of loading buffer) was injected in sample order one batch at a time, with one sample from each batch randomly selected and acquired twice. Identical LC conditions were used as described above, with *m*/*z* window sizes determined based on precursor *m*/*z* frequencies in previous IDA data. SWATH variable window acquisition with a set of 100 overlapping windows (1 amu for the window overlap) was constructed covering the mass range of *m*/*z* 399.5–1249.5. In SWATH mode, first a TOFMS survey scan was acquired (*m*/*z* 350–1500, 0.05 s) then the 100 predefined *m*/*z* ranges were sequentially subjected to MS/MS analysis. Product ion spectra were accumulated for 30 ms in the mass range *m*/*z* 350–1500 with rolling collision energy optimised for lowed *m*/*z* in *m*/*z* window +10%, with the total duty cycle of 3.09 s.

SWATH-MS Quality Control—One sample from each batch was randomly selected and acquired twice. In addition, a commercially available human plasma which was used as a control plasma standard sample (ST01) was acquired once at the beginning, the pooled sample acquired in duplicate once at the beginning and once at the end of the series. Quality control samples and technical replicate samples were run and extracted together with the samples to assess the overall data quality. Total area normalisation was applied. Data quality was evaluated using PCA, and CV was calculated for all quality control samples and technical replicates.

### SWATH data analysis

SWATH peaks were extracted using PeakView (v.2.2). Retention times of peptides between the 2D‐IDA and SWATH analysis were aligned with the aid of endogenous peptides using the retention time alignment feature of PeakView software. The Shared and modified peptides were excluded. Peak extraction parameters were set as the following: 100 peptides per protein, 6 transition ions per peptide, peptide confidence threshold 99%, FDR extraction threshold 1%, XIC (Extract Ion Chromatogram) retention time window 10 min and mass tolerance 75 ppm. The extracted transition ion peak areas, peptide peak areas and protein peak areas were exported in Excel for further statistical analysis.

### Statistical analysis

Quantitative MS data was obtained from 54 independent biological samples for the three groups (20 Health, 5 COVID-19 ARDS and 29 MIS-C). Statistical analysis was performed using similar methods that are described in^39^. In brief, the peptide ion peak areas were averaged for the replicate technical injections, then further scaled so that the sum of the ion intensities of each sample equal the maximum total ion intensity. The normalised ion intensities were summed for each peptide and protein. The entire protein level data was clustered using hierarchical clustering (correlation distance and complete linkage) and visualised using heatmaps. Multivariate analysis such as PCA was used to examine global trends among sample groups.

Two approaches were undertaken to determine differential expression between groups: an analysis of variance on the log-transformed normalised protein peak areas, and un-paired *t* test between individual groups. For the analysis of variance, proteins were deemed to be differentially expressed if the ANOVA *p* value was less than 0.05, and the maximum protein fold change exceeded 1.5. Rather than adjusting the ANOVA *p* values for multiple testing using the false-discovery rate criterion of Benjamini and Hochberg, we used a combination of un-corrected *p* value with a fold change threshold. This approach has been shown to represent a higher number of true-positives with an acceptably low quantitative false-discovery rate^40^. Additional adjustment of results creates a criterion that is too stringent and creates a risk of excluding true positives. For the individual comparisons, samples from all selected pairs of conditions were compared first by a two-sample *t* test of the log-transformed protein areas, and secondly by combining individual peptide-level ratios for each protein. The reporting threshold required a *t* test *p* value less than 0.05 and fold change exceeding 1.5.

### Pathway analysis

Differentially expressed proteins were examined in the context of biological data using the Reactome Over-representation Pathway Analyses tool^41^ and KEGG pathways analysis of STRING software [v10.5, http://string-db.org^42^,], respectively, for determining the relevant pathway enrichments. Pathways identified were ranked by *p* value.

### Reporting summary

Further information on research design is available in the Nature Research Reporting Summary linked to this article.

## Supplementary information

Description of Additional Supplementary Files

Supplementary Data 1

Supplementary Data 2

Supplementary Data 3

Supplementary Data 4

Reporting Summary

## Data Availability

The raw complete mass spectrometry proteomics data generated in this study been deposited to the ProteomeXchange Consortium database via the PRIDE partner repository with the dataset identifier PXD025125. This can be accessed using the following link (https://www.ebi.ac.uk/pride/archive/projects/PXD025125). The SwissProt identifiers that support the data in this manuscript are publicly available from the uniprot database https://www.uniprot.org/uniprot/?query=reviewed:yes and FASTA file can be downloaded https://ftp.uniprot.org/pub/databases/uniprot/current_release/knowledgebase/complete/uniprot_sprot.fasta.gz. Supplementary data 2–4 report specific Uniprot Accession IDs for each protein discovered in this manuscript. These uniprot accession IDs were used for further pathway analysis. Pathway analysis in this study is based on publicly available databases accessible from https://reactome.org/ where uniprot accession to pathways can be downloaded https://reactome.org/download/current/UniProt2Reactome.txt, and https://string-db.org/ where full database can be accessed directly from https://stringdb-static.org/download/items_schema.v11.5.sql.gz.

## References

[1] Dong E, Du H, Gardner L (2020). An interactive web-based dashboard to track COVID-19 in real time. Lancet Infect. Dis..

[2] The Novel Coronavirus Pneumonia Emergency Response Epidemiology Team. (2020). The epidemiological characteristics of an outbreak of 2019 novel coronavirus diseases (COVID-19)—China, 2020. China CDC Wkly..

[3] Singhal T. A review of coronavirus disease-2019 (COVID-19). Indian J. Pediatr. **87**, 1–6 (2020). 10.1007/s12098-020-03263-6 PMC7090728 32166607

[4] Rothe C (2020). Transmission of 2019-nCoV infection from an asymptomatic contact in Germany. N. Engl. J. Med..

[5] Huang C (2020). Clinical features of patients infected with 2019 novel coronavirus in Wuhan, China. Lancet.

[6] Zhou F (2020). Clinical course and risk factors for mortality of adult inpatients with COVID-19 in Wuhan, China: a retrospective cohort study. Lancet.

[7] World Health Organisation. Report of the WHO-China Joint Mission on Coronavirus Disease 2019 (COVID-19). (WHO, 2020).

[8] Lu X (2020). SARS-CoV-2 infection in children. N. Engl. J. Med..

[9] Bialek S (2020). Coronavirus Disease 2019 in Children—United States, February 12–April 2, 2020. Morbid. Mortal. Wkly. Rep..

[10] World Health Organization. Novel coronavirus. http://www.who.int/csr/don/12-january-2020-novel-coronavirus-china/en/ (2020).

[11] Jiehao C (2020). A case series of children with 2019 novel coronavirus infection: clinical and epidemiological features. Clin. Infect. Dis..

[12] Riphagen S, Gomez X, Gonzalez-Martinez C, Wilkinson N, Theocharis P (2020). Hyperinflammatory shock in children during COVID-19 pandemic. Lancet.

[13] Jones VG (2020). COVID-19 and Kawasaki disease: novel virus and novel case. Hosp. Pediatr..

[14] Verdoni L (2020). An outbreak of severe Kawasaki-like disease at the Italian epicentre of the SARS-CoV-2 epidemic: an observational cohort study. Lancet.

[15] Grimaud M (2020). Acute myocarditis and multisystem inflammatory emerging disease following SARS-CoV-2 infection in critically ill children. Ann. Intensive Care.

[16] Whittaker E. et al. Clinical characteristics of 58 children with a pediatric inflammatory multisystem syndrome temporally associated with SARS-CoV-2. *JAMA*. **324**, 259–269 (2020). 10.1001/jama.2020.10369 PMC7281356 32511692

[17] Capone CA (2020). Characteristics, cardiac involvement, and outcomes of multisystem inflammatory syndrome of childhood associated with severe acute respiratory syndrome coronavirus 2 Infection. J. Pediatr..

[18] Paediatrics RCo, Health C. Guidance-Paediatric multisystem inflammatory syndrome temporally associated with COVID-19. College Reino Unido (2020).

[19] Pouletty M (2020). Paediatric multisystem inflammatory syndrome temporally associated with SARS-CoV-2 mimicking Kawasaki disease (Kawa-COVID-19): a multicentre cohort. Ann. Rheum. Dis..

[20] Toubiana, J. et al. Kawasaki-like multisystem inflammatory syndrome in children during the covid-19 pandemic in Paris, France: prospective observational study. BMJ. **369**, m2094 (2020). 10.1136/bmj.m2094 PMC7500538 32493739

[21] Belhadjer Z (2020). Acute heart failure in multisystem inflammatory syndrome in children in the context of global SARS-CoV-2 pandemic. Circulation.

[22] Ramcharan T., et al. Paediatric inflammatory multisystem syndrome: temporally associated with SARS-CoV-2 (PIMS-TS): cardiac features, management and short-term outcomes at a UK tertiary paediatric hospital. *Pediatr Cardiol*. **41**, 1391–1401 (2020). 10.1007/s00246-020-02391-2 PMC7289638 32529358

[23] Messner, C. B. et al. Ultra-high-throughput clinical proteomics reveals classifiers of COVID-19 infection. *Cell Syst*. **11**, 11–24 (2020). 10.1016/j.cels.2020.05.012 PMC7264033 32619549

[24] Shen B (2020). Proteomic and metabolomic characterization of COVID-19 patient sera. Cell.

[25] Diorio C (2020). Evidence of thrombotic microangiopathy in children with SARS-CoV-2 across the spectrum of clinical presentations. Blood Adv..

[26] Matsushita M (2002). Activation of the lectin complement pathway by H-ficolin (Hakata antigen). J. Immunol..

[27] Holter JC (2020). Systemic complement activation is associated with respiratory failure in COVID-19 hospitalized patients. Proc. Natl Acad. Sci..

[28] Al‐Ghafry M (2020). Are children with SARS‐CoV‐2 infection at high risk for thrombosis? Viscoelastic testing and coagulation profiles in a case series of pediatric patients. Pediatr. Blood Cancer.

[29] Tang N, Li D, Wang X, Sun Z (2020). Abnormal coagulation parameters are associated with poor prognosis in patients with novel coronavirus pneumonia. J. Thromb. Haemost..

[30] Nagelkerke SQ, Kuijpers TW (2015). Immunomodulation by IVIg and the role of FC-gamma receptors: classic mechanisms of action after all?. Front. Immunol..

[31] Harwood, R. et al. A national consensus management pathway for paediatric inflammatory multisystem syndrome temporally associated with COVID-19 (PIMS-TS): results of a national Delphi process. *Lancet Child Adolesc Health.* **5**, 133–141 (2021). 10.1016/S2352-4642(20)30304-7 PMC7500943 32956615

[32] Paoli M, Marles-Wright J, Smith A (2002). Structure–function relationships in heme-proteins. DNA Cell Biol..

[33] Ascenzi P (2005). Hemoglobin and heme scavenging. IUBMB Life.

[34] Gaggar A, Patel RP (2016). There is blood in the water: hemolysis, hemoglobin, and heme in acute lung injury. Am. J. Physiol. Lung Cell Mol. Physiol..

[35] Sarohan AR (2020). COVID-19: endogenous retinoic acid theory and retinoic acid depletion syndrome. Med. Hypotheses.

[36] Kell AM, Gale M (2015). RIG-I in RNA virus recognition. Virology.

[37] Dowell SF (1996). Treatment of respiratory syncytial virus infection with vitamin A: a randomized, placebo-controlled trial in Santiago, Chile. Pediatr. Infect. Dis. J..

[38] Pediatric acute respiratory distress syndrome: consensus recommendations from the Pediatric Acute Lung Injury Consensus Conference. Pediatr. Crit Care Med. **16**:428–439 (2015). 10.1097/PCC.0000000000000350 PMC5253180 25647235

[39] Hoq M (2019). A prospective, cross-sectional study to establish age-specific reference intervals for neonates and children in the setting of clinical biochemistry, immunology and haematology: the HAPPI Kids study protocol. BMJ Open.

[40] Wu JX (2016). SWATH mass spectrometry performance using extended peptide MS/MS assay libraries. Mol. Cell Proteom..

[41] Fabregat A (2017). Reactome pathway analysis: a high-performance in-memory approach. BMC Bioinform..

[42] Szklarczyk D (2015). STRING v10: protein-protein interaction networks, integrated over the tree of life. Nucleic Acids Res..

