# Supplementary material for: Pathophysiological pathway differences in children who present with COVID-19 ARDS compared to COVID -19 induced MIS-C
Source: Nat Commun. 2022 May 2;13:2391. doi: 10.1038/s41467-022-29951-9 (PMC9061738; doi:10.1038/s41467-022-29951-9)
Supplement: Supplementary file 1 — Description of Additional Supplementary Files [file 41467_2022_29951_MOESM1_ESM.docx]

File Name: Supplementary Data 1

Description: Sampling, Diagnosis, Intravenous Immunoglobulin (IVIG) and Steroid administration for COVID-19 patients with MIS-C and COVID-19 patients with ARDS.

File Name: Supplementary Data 2

Description: ANOVA analysis showing relative protein expression across the Healthy, COVID-19 ARDS and MIS-C Groups. *UniProt Knowledge Base Identification number; #MaxFC for Ratio of largest / lowest group average; ‡p-values for ANOVA analysis; ❐ Adjusted p-values for ANOVA analysis using benjamini-hochberg adjustment. Bolded results are statistically significant. Results are expressed as mean variation in protein expression.

File Name: Supplementary Data 3

Description: Student’s T-test showing the variation in protein expression between COVID-19 ARDS Group and Healthy Group. *UniProt Knowledge Base Identification number; Fold change for the ratio of relative proteins intensities values between Healthy Group and COVID-19 ARDS Group; ‡p-values for Student’s T-test analysis; ❐ Adjusted p-values for ANOVA analysis using benjamini-hochberg adjustment. Bolded results are statistically significant. Results are expressed as mean variation in protein expression.

File Name: Supplementary Data 4

Description: Student’s T-test showing the variation in protein expression between MIS-C Group and Healthy Group. *UniProt Knowledge Base Identification number; Fold change for the ratio of relative proteins intensities values between Healthy Group and MIS-C Group; ‡p-values for Student’s T-test analysis; ❐ Adjusted p-values for ANOVA analysis using benjamini-hochberg adjustment. Bolded results are statistically significant. Results are expressed as mean variation in protein expression.
