# Supplementary material for: Pathophysiological pathway differences in children who present with COVID-19 ARDS compared to COVID -19 induced MIS-C
Source: Nat Commun. 2022 May 2;13:2391. doi: 10.1038/s41467-022-29951-9 (PMC9061738; doi:10.1038/s41467-022-29951-9)
Supplement: Supplementary file 6 — Reporting Summary [file 41467_2022_29951_MOESM6_ESM.pdf]

nature portfolio

Corresponding author(s): Professor Vera Ignjatovic

Last updated by author(s): Apr 2, 2022

Reporting Summary

Nature Portfolio wishes to improve the reproducibility of the work that we publish. This form provides structure for consistency and transparency in reporting. For further information on Nature Portfolio policies, see our Editorial Policies and the Editorial Policy Checklist.

Statistics

For all statistical analyses, confirm that the following items are present in the figure legend, table legend, main text, or Methods section.

n/a	Confirmed	
☐	☒	The exact sample size (n) for each experimental group/condition, given as a discrete number and unit of measurement
☐	☒	A statement on whether measurements were taken from distinct samples or whether the same sample was measured repeatedly
☐	☒	The statistical test(s) used AND whether they are one- or two-sided<br>*Only common tests should be described solely by name; describe more complex techniques in the Methods section.*
☐	☐	A description of all covariates tested
☐	☒	A description of any assumptions or corrections, such as tests of normality and adjustment for multiple comparisons
☐	☒	A full description of the statistical parameters including central tendency (e.g. means) or other basic estimates (e.g. regression coefficient) AND variation (e.g. standard deviation) or associated estimates of uncertainty (e.g. confidence intervals)
☐	☒	For null hypothesis testing, the test statistic (e.g. F, t, r) with confidence intervals, effect sizes, degrees of freedom and P value noted<br>*Give P values as exact values whenever suitable.*
☒	☐	For Bayesian analysis, information on the choice of priors and Markov chain Monte Carlo settings
☐	☒	For hierarchical and complex designs, identification of the appropriate level for tests and full reporting of outcomes
☒	☐	Estimates of effect sizes (e.g. Cohen's d, Pearson's r), indicating how they were calculated

Our web collection on statistics for biologists contains articles on many of the points above.

Software and code

Policy information about availability of computer code

Data collection	No Software included in data collection.
Data analysis	Proteins were identified using ProteinPilot (v5.0, Sciex). Data were analysed using PeakView v.2.2 SciEx and R

For manuscripts utilizing custom algorithms or software that are central to the research but not yet described in published literature, software must be made available to editors and reviewers. We strongly encourage code deposition in a community repository (e.g. GitHub). See the Nature Portfolio guidelines for submitting code & software for further information.

Data

Policy information about availability of data

All manuscripts must include a data availability statement. This statement should provide the following information, where applicable:

- Accession codes, unique identifiers, or web links for publicly available datasets
- A description of any restrictions on data availability
- For clinical datasets or third party data, please ensure that the statement adheres to our policy

The mass spectrometry proteomics data have been deposited to the ProteomeXchange Consortium via the PRIDE partner repository with the dataset identifier PXD025125. This can be accessed using the following link (https://www.ebi.ac.uk/pride/archive/projects/PXD025125)
The SwissProt identifiers that support the data in this manuscript are publicly available from https://www.uniprot.org/uniprot/?query=reviewed:yes. Pathway analysis in this study is based on publicly available databases accessible from https://reactome.org/ and https://string-db.org/.

Field-specific reporting

Please select the one below that is the best fit for your research. If you are not sure, read the appropriate sections before making your selection.

- [x] Life sciences
- [ ] Behavioural & social sciences
- [ ] Ecological, evolutionary & environmental sciences

For a reference copy of the document with all sections, see nature.com/documents/nr-reporting-summary-flat.pdf

Life sciences study design

All studies must disclose on these points even when the disclosure is negative.

Sample size	Sample size was determined based on the limited number of children presenting with MIS-C and COVID-19 ARDS. This is extremely low in the population. This has been clearly discussed as a limitation within the manuscript.
Data exclusions	No data excluded.
Replication	This study does not include biological replicates. Technical replicates and quality control were completed at the Mass-Spectromety data acquisition stage. One sample from each batch was randomly selected and acquired twice, as well as samples pooled from one batch in data acquisition and data analysis for assessing data quality in each batch.
Randomization	Allocation to groups was not random and was determined based on a diagnostic criteria (whether diagnosed with MIS-C or COVID-19 ARDS).
Blinding	Samples were organised and batched randomly across multiple batches into the mass-spectrometer. Samples were de-identified to a random study ID at the mass spectrometry analysis stage and groups were not known to the technician. Samples were not classified as "healthy", "MIS-C" or "ARDS" until statistical comparison of groups occurred.

Reporting for specific materials, systems and methods

We require information from authors about some types of materials, experimental systems and methods used in many studies. Here, indicate whether each material, system or method listed is relevant to your study. If you are not sure if a list item applies to your research, read the appropriate section before selecting a response.

Materials & experimental systems

n/a	Involved in the study	
x		Antibodies
x		Eukaryotic cell lines
x		Palaeontology and archaeology
x		Animals and other organisms
	x	Human research participants
	x	Clinical data
x		Dual use research of concern

Methods

n/a	Involved in the study	
x		ChIP-seq
x		Flow cytometry
x		MRI-based neuroimaging

Human research participants

Policy information about studies involving human research participants

Population characteristics	We enrolled three populations into this study.<br>MIS-C: n = 29, 13M/16F, 4.56 ± 2.56 years old. No additional demographic details are collected or reported in this study.<br>COVID-19 ARDS: n= 5, 3M/2F, 9.38 ± 3.04, No additional demographic details are collected or reported in this study.<br>Healthy Children: n=20, 12M/8F, 8.42 ± 6.51. No additional demographic details are collected or reported in this study.
Recruitment	MIS-C and COVID-19 ARDS groups were enrolled at Hospital Necker, France when a child presented at the hospital with either MIS-C or COVID-19 ARDS phenotype. Confirmation of COVID-19 diagnosis was by PCR, serological testing or CT scan. ARDS diagnoses were based on Pediatric Acute Lung Injury Consensus Conference Group for the diagnosis of ARDS in COVID19 patients and little bias is expected diagnosing this group.<br>MIS-C was diagnosed based on patients having a positive COVID-19 test and having three of the following symptoms: cervical lymphadenopathy, bulbar conjunctivitis, skin rash, erythema of oral and pharyngeal mucosa, gastrointestinal symptoms (abdominal pain, diarrhea, vomiting), asthenia, meningismus, respiratory signs, heart failure, or cardiogenic shock. As these symptoms are quite distinct from typical COVID-19 and we require presence of 3, we do not expect bias to impact these results.<br>Healthy samples were collected and stored prior to the commencement of the COVID-19 pandemic and as such could not be possible for COVID-19.
Ethics oversight	This study was approved by the Royal Children's Hospital Ethics in Human Research Committee, reference number 34184; and the Necker Hospital, France, Assistance Publique - Hôpitaux de Paris reference number APHP- registration N°.2020 0428163907 and Trial Registration: NCT04420468.

Note that full information on the approval of the study protocol must also be provided in the manuscript.

Clinical data

Policy information about clinical studies

All manuscripts should comply with the ICMJEguidelines for publication of clinical research and a completedCONSORT checklist must be included with all submissions.

Clinical trial registration	This study incorporated samples collected from the study with Clinical Trial Registration NCT04420468
Study protocol	Full trial protocol can be accessed https://clinicaltrials.gov/ct2/show/NCT04420468
Data collection	The study consists an extensive retrospective data collection from April 2020 till the end of the main SARS-Cov-2 outbreak, from medical file of children have exhibited acute myocarditis and cared in 4 pediatric intensive care units of Assistance Publique-Hôpitaux de Paris of Ile-de-France region.<br>Children presented with an acute myocarditis, fever and shock with a possible COVID-19 infection cared between April 2020 till the end of the main SARS-Cov-2 outbreak in 4 AP-HP Parisian hospitals :<br><br>Necker-Enfants Malades<br>Armand Trousseau<br>Robert Debré<br>Kremlin Bicêtre
Outcomes	Primary Outcome Measures :<br>Acute myocarditis [Time Frame: 7 days]<br>Occurrence, description and time course of acute myocarditis<br><br>Multi-systemic inflammatory syndrome [Time Frame: 7 days]<br>Occurrence, description and time course of multi-systemic inflammatory syndrome, features of Kawasaki disease<br><br>Kawasaki disease [Time Frame: 7 days]<br>Occurrence, description and time course of features of Kawasaki disease<br><br>Secondary Outcome Measures :<br>Results of SARS-CoV-2 [Time Frame: 7 days]<br>Results of SARS-CoV-2 screening either by PCR or antibodies serological assay
